# Foundlings

**Published:** 1871-07

**Authors:** 


### FOUNDLINGS.

Vice and crime and disease go together, and come by inheritance. Three out of four of all infants "found" in the streets, and more, die before one year of age. Only one person out of thirteen hundred of the inhabitants of France, come under legal penalty for wrong doing; one out of every hundred and fifty-eight foundlings finds its way to prison. This results partly from inherited moral taint, and partly from the absence of a mother's love and care, and want of opportunities of training and instruction inseparable from mere hireling attentions.

Immense amounts of money have been expended in building large asylums, and procuring medical aid and proper nurses for the little unfortunates, but it is a waste of money which would accomplish an immeasurable good if differently appropriated. With all the care and attention which are bestowed in these establishments, three fourths of the children die in this country, and a like result is manifested in the large cities of the Old World, notwithstanding the accumulated experiences of generations.

A large number of sick persons in any one building is unfavorable to recovery. The true method of caring for the sick is to divide them out among farmers, one or two at a house; if one child was partitioned out to each family, the mortality among foundlings would be diminished more than one half. In a district of Massachusetts where seventy-five out of a hundred sick children died, a lady of great intelligence and force of character took the matter in hand, and in the course of a very few years only ten children out of a hundred died, instead of seventy or eighty. Let the care of foundlings be intrusted to intelligent, conscientious Christian families of very moderate means, with a reasonable compensation, less per head, a great deal, than the present foundling system costs, and not only will the mortality be diminished forty per cent. and even more, but by reason of the Christian education and surroundings, not one in thirteen hundred will find its way into a penitentiary, — not one in many thousands.

No human laws can prevent illegitimacy. In European countries, the most civilized, the most cultivated, one third of those born are foundlings; thirty in a hundred in New York; and in Philadelphia only four in a hundred; but in the progress of "civilization," as it is called, by which the few rich become richer, and the many poor become poorer, this proportion will fearfully increase, and no human laws can prevent it; nothing upon earth can stay the increase except a high and wide spreading CHRISTIAN SENTIMENT.

